# Maintenance of Dental Records and Forensic Odontology Awareness: A Survey of Croatian Dentists with Implications for Dental Education

**DOI:** 10.3390/dj9040037

**Published:** 2021-03-25

**Authors:** Ivana Savić Pavičin, Ana Jonjić, Ivana Maretić, Jelena Dumančić, Ajla Zymber Çeshko

**Affiliations:** 1 Department of Dental Anthropology, School of Dental Medicine, University of Zagreb, 10000 Zagreb, Croatia; savic@sfzg.hr; 2 School of Dental Medicine, University of Zagreb, 10000 Zagreb, Croatia; ana.jonjic94@gmail.com; 3 Private Dental Polyclinic Vinica, 42000 Varaždin, Croatia; s.mareticivana@gmail.com; 4 Department of Dental Medicine, University Hospital Centre Zagreb, 10000 Zagreb, Croatia; 5 School of Dentistry, FAMA College, 10000 Prishtina, Kosovo; ajlazymber1@icloud.com

**Keywords:** dental record, record keeping, documentation, forensic odontology, dental education, Croatia

## Abstract

Forensic odontology is the application of dentistry within the criminal justice system. Forensic expertise, including dental identification, mostly relies on dental records. We explored the practice of maintaining dental records among Croatian dentists, as well as their knowledge of legal regulations and the application of dental records in forensic odontology. In all, 145 dentists participated in an online survey. Questions covered general information on dentists, maintenance of dental records, and knowledge of legal requirements and forensic odontology. Overall, 70% of dentists obtain and archive written informed consents, while 87% record dental status. Generally, non-carious dental lesions and developmental dental anomalies were not recorded. About 72% of dentists record filling material and surfaces. Only 32% of dentists know the legal requirements for keeping records, whereas 21% have no knowledge of forensic odontology and its purpose. The survey revealed different practices in the maintenance of dental records, including significant flaws and lack of awareness of its forensic importance. This obvious need for additional education on proper maintenance of dental records could be met by including forensic odontology in compulsory undergraduate courses and postgraduate dental education. Establishing national and international standards in dental charting would comply with contemporary trends in health care and the requirements of forensic expertise.

## 1. Introduction

Forensic odontology is a branch of dentistry that applies dental science in order to provide evidence in the interest of the law [1]. It includes dental identification, bitemark analysis, age estimation, and expertise in civil litigation cases related to dental malpractice and injuries [2]. Besides dental identification, other forensic odontology methods can be utilized in personal identification, such as lip prints (cheiloscopy) and palatal rugae patterns (rugoscopy) [3,4]. Most of these procedures rely on dental documentation, which is a source regarding an individual’s antemortem dental information. It consists of their dental record with a status chart, intraoral and extraoral X-rays, photographs, dental casts, medical history, and written consent [5]. Numerous qualitative and quantitative characteristics of teeth make dental identification a high-value forensic procedure. Its advantages are the ease of utilizing such resources as well as minor technological and financial requirements. Dental identification is especially useful in cases where teeth are the only remaining preserved parts of a human body: fires, mass graves, plane crashes, or natural disasters such as floods and avalanches [5,6,7,8]. However, for dental identification to be successful, dentists must conscientiously keep dental documentation on their patients. Additionally, the importance of maintaining and keeping dental records in terms of legal requirements should also be emphasized, where such records demonstrate the quality and thoroughness of dental care when presented in court proceedings and when providing forensic expertise [5]. Maintaining and keeping dental records are legal obligations for all dentists [9].

In Croatia, the use of dental identification began in the 1970s, when mass casualties occurred in two traffic accidents. Later, another significant development was during and after the Croatian War of Independence 1991–1995, when a need to identify victims from mass graves emerged [10].

The lack of data on the current practice of maintaining dental records has led us to investigate the thoroughness and comprehensiveness of practices in maintaining dental records, including knowledge and awareness among dentists of the legal importance and possibilities of using such records for forensic purposes.

## 2. Materials and Methods

This research was conducted using an online questionnaire (Google Forms) and titled “Questionnaire on Practices and Quality of Maintaining Dental Records in the Republic of Croatia and Possibility of its Use for Forensic Purposes”. Prior to its use, it received approval from the Ethics Committee of the School of Dental Medicine, University of Zagreb. In 2019, the questionnaire was sent to 197 email addresses, available publicly or through social media. In all, 145 dentists participated in the survey, giving a response rate of 74%.

The survey consisted of 40 questions with single, multiple choice, and open-ended questions.

Given below is an outline of the organization of the questions into five sections.

### 2.1. General Information on Dentists

The first group of questions was formulated to obtain information on gender, age, years of work experience, and the location of the respondent’s dental practice. This included questions on the school of basic dental degree and type of employment covering options such as public dental service at a health center, private practice under contract with the Croatian Health Insurance Fund (CHIF), exclusively private practice, or working in a clinic or polyclinic.

### 2.2. Data on Dental Documentation

The questions focused on the practice of taking the general patient information such as gender, date of birth, contact number, and address, as well as the maiden name for female patients, names of other dentists the patient has visited, and name of their general practitioner.

Questions also covered recording medical history and entering dental status in the patient’s records. This also included information on trauma, anomalies in tooth number, position and morphology, and developmental changes in dentitions. The issue of using abbreviations in documentation and treatment codes as stipulated by the CHIF was also addressed. The frequency of the routine use of X-rays and photographs was also examined.

### 2.3. Information on Dental Documentation Keeping

Questions posed in this section addressed the format of dental documentation, including all relevant components and the duration of storing dental documentation as stipulated in the Dental Medicine Act of the Republic of Croatia [9]. A question was also asked as to what dentists considered a barrier to better management of dental documentation in their everyday work

### 2.4. Knowledge of Legal Aspects of Dental Practice

Respondents were given the opportunity to answer questions on obtaining written consents, archiving them, and their knowledge of the right of patients to dispose of personal dental records.

### 2.5. Awareness of Forensic Odontology

This section enquired about the level of awareness among dentists and their acquired education in forensic odontology.

A statistical analysis was performed using IBM SPSS Statistics (Version 25.0. Armonk, NY, USA: IBM Corp.). A chi-square test was used to assess differences in categorical variables except in cases when there were less than 10 participants per cell, when Fisher’s exact test was used. The statistical significance level was set at *p* < 0.05.

## 3. Results

### 3.1. General Information on Dentists 

The largest number of respondents belonged to the age group of 25 to 45 years, possessed 5 to 20 years of work experience, and were employed in a public health center, respectively. Most of the respondents were graduates of the School of Dental Medicine, University of Zagreb, and were employed in cities across Central Croatia. (Table 1).

### 3.2. Data on Dental Documentation 

Statistical analysis shows that 86.9% of dentists record dental status at first visit, and this routine is more prevalent among dentists in health centers and private practices operating under a CHIF contract than those operating exclusively in private practices (*p* < 0.05). (Table 2).

The practice of updating dental status data is less common for male than female dentists (*p* < 0.05). Dentists in Istria collect significantly more additional data on patients compared with dentists in Dalmatia (*p* < 0.05). In collecting additional data on patients, dentists employed in clinics or polyclinics are more up to date than employees in private practices (*p* < 0.05). When recording details for restorative treatment, such as the color of a material, Black’s classification, or the type of preparation, female dentists recorded more details than their male counterparts (*p* < 0.05). The analysis also shows that dentists employed in health centers more often keep old codes for treatment as stipulated by the CHIF than doctors employed in private practices operating under a CHIF contract (*p* < 0.05).

Extraoral and intraoral photographs are more often used by doctors in Central Croatia and Istria than by doctors in Slavonia (*p* < 0.05). Also, photos are used more often in private surgeries, whether operating under a CHIF contract or not, and in clinics, than in health centers (*p* < 0.05).

### 3.3. Data on Dental Documentation Keeping 

The largest number of respondents answered that they keep dental records for a period of five to ten years (33%) while a further 45% keep such records for even longer.

The analysis shows that documentation was kept longer by doctors in Istria and the Croatian Littoral (northern coastal region) than in Dalmatia (southern coastal region) and Slavonia (*p* < 0.05). The same practice is observed in doctors employed in clinics and polyclinics compared with dentists employed in health centers or private practices operating under a CHIF contract (*p* < 0.05). Statistical analysis shows that dentists in health centers kept X-rays for a significantly shorter period (less than five years) compared with employees in private practices operating under a CHIF contract (*p* < 0.05). On the other hand, it is evident that doctors in private surgeries, regardless of whether operating under a CHIF contract or not, keep X-rays for significantly longer (more than 20 years) compared with those in health centers (*p* < 0.05). (Table 3).

### 3.4. Knowledge of Legal Aspects of Dental Practice 

In all, 69% of dentists seek written consents prior to treatment, with a significantly larger number in Istria than those in Dalmatia (*p* < 0.05). Depending on the type of dental practice, the analysis showed that doctors employed in clinics and polyclinics are more likely to obtain written consents than doctors employed in health centers and private practices (*p* < 0.05). (Table 4).

### 3.5. Awareness of Forensic Odontology 

Respondents who obtained their degree in dentistry outside of Croatia more often answered that they have no education or training in forensic odontology (*p* < 0.05). (Table 5).

## 4. Discussion

The total number of collected responses to the questionnaire was 145, equivalent to a response rate of 74%. Accordingly, the survey sampled 2.86% of the total number of currently active dentists in the Republic of Croatia, according to data from the Croatian Dental Chamber [11]. Although it is a low proportion of the total target population, the number is consistent with responses in similar studies [1,12,13]. Our study is the first study on the manner and quality (suitability) of maintaining dental records in the Republic of Croatia. Dentists from all parts of Croatia participated in the survey, with the highest response coming from Central Croatia, followed by Dalmatia. This is due to the larger population of cities in those areas, especially Zagreb as the Croatian capital and Split. The largest number of respondents coming from the 25 to 45 age group can be explained by the higher digital literacy of the younger generation of dentists.

Table 6 lists the current legal requirements for dental documentation maintenance in Croatia. Based on the results obtained in this study, it is evident that Croatian dentists keep dental records in line with statutory requirements and record basic patient data, such as gender, date of birth, address, and contact phone number. About 46% of respondents regularly entered additional data, which can be useful in forensic procedures. This was the case significantly more often for dentists in Istria and the Croatian Littoral as well as those working in clinics and polyclinics. This may be due to greater development of dental tourism in Istria and the Littoral, where foreign patients are treated [14], and possibly a greater awareness among dentists of the need to establish protective measures against potential lawsuits.

Dental charting, handwritten or electronic, should provide an up-to-date insight into the status of a patient’s dentition, i.e., the number of existing natural teeth, detected caries lesions, fixed or mobile prosthetic works, fillings, and extractions. During a patient’s first visit, dental status is taken by 87% of respondents, more often dentists working in health centers and private practices under a CHIF contract, compared with exclusively private practices (*p* < 0.05). A possible reason for this may be the fact that patients visit public or private surgeries operating under a CHIF contract over a longer period of time, and also when requiring conservative treatment, which is mainly covered by the CHIF, whereas patients visit private dental practice only for specific treatment or procedures, which are usually not covered by the CHIF. The second reason is that the CHIF supervises and controls the work of contracted practices.

Dental status changes are entered in status charts by 31% of dentists, with 28% of them doing so twice a year, which may correspond to the term “each visit”, as regular dental checkups are usually performed every six months. Therefore, almost 60% of dentists regularly update dental charts. Although the proportion of dentists who do not record a detailed status at the first patient visit is small (13%), noting that approximately 40% do not update such statuses regularly, these data are somewhat worrying. In terms of children’s oral health care, regularly recording changes in dentition is important, and was performed by 55% of dentists in our study. In forensic analysis, developmental changes in dentition enable estimation of dental age. As for children, there can be only a small number of restorations, if any, and dental age estimation may be crucial for individual identification. An example of such a case is the plane crash over Vrbovec (Croatia), which happened in 1976, where dental age estimation provided supportive evidence for the identification of eight child victims of the accident [10].

Dental anomalies are also important for dental identification because they are relatively rare and provide a unique characteristic to dentition without caries and dental procedures. As the incidence of caries decreases in highly developed countries, the importance of anomalies for dental identification will increase even more. This study shows that dentists rarely recorded dental anomalies except for tooth number anomalies, which were recorded by 60% of respondents (Table 2). Also, only 35% of respondents recorded non-carious dental lesions such as erosion, attrition, and abrasion.

After performing a restorative procedure, data on materials and methods should be entered in the progress notes in the patient record. The majority of surveyed dentists, about 70%, use abbreviations or codes for recording treatment, while only 30% keep old lists of abbreviations after changes. This is a worrying fact because analysis of premortem dental data requires legible, clear, and easily accessible information [1,15]. Statistical analysis has shown that keeping old codes was more often done by dentists who practice in health centers. The reason for this is the fact that dentists in health centers use codes for the purpose of charging of fees to the CHIF, while in private practices payments are made by patients.

For tooth notation, 72% of dentists most often use the FDI tooth numbering system, while the Palmer–Zsigmondy system is more commonly used by respondents who graduated from the University of Split.

The most common choice of radiological image for a routine check-up was an orthopantomogram (95%). Five respondents (3%) answered that they do not routinely take X-rays. Although this is small in number, it is a warning which indicates insufficient education on the importance of X-rays in diagnosing various conditions otherwise undetectable in clinical examinations, as well as in planning therapy and monitoring development in children.

Though not obligatory except in orthodontics and oral rehabilitation, intraoral and extraoral photographs are an excellent complementary record for dental documentation and have great importance in forensics. According to the survey results, photographs were used by 61% of respondents. We consider this a good result, given that it is optional in dental documentation. In modern private practices, dental photography is increasingly used in documenting the initial status of patients and therapy planning.

Most of the respondents (93%) keep documentation in digital form, with 73% using storage on additional media as protection against alteration, premature destruction, or unauthorized use, as required by law [9]. The duration of archiving X-rays was most often 5 to 10 years (37%), with a further 40% of respondents archiving X-rays for even longer. Comparing antemortem and postmortem radiographs may be crucial for dental identification. Even old radiographs can be used to compare tooth morphology and surrounding bone structures.

More than 94% of respondents keep dental casts and temporary replacements in dental documentation. Serial numbers of implants are recorded by 61% of respondents. Statistical analysis showed that dentists working in private practices keep implant serial numbers for significantly longer than dentists in health centers. The reason may be that most implants are used in private surgeries since the CHIF does not cover the costs of implant treatment. Another explanation may be the high cost of implants and possible complaints.

In our study, when asked about barriers to better record keeping, 76% of respondents said it was a lack of time, while 30% indicated a lack of education.

The obligation to keep dental records in Croatia is regulated by the Dental Medicine Act [9]. Only 57 respondents (39%) answered that they know about the period that the law prescribes for archiving documentation, and only 47 (32%) gave the correct answer of 10 years. Most respondents acquired training in forensic dentistry in their undergraduate studies (72%), with more than a fifth of respondents stating that they have no knowledge about forensic odontology and its purpose. Statistical analysis shows that most dentists lacking knowledge of forensic odontology studied outside Croatia (abroad).

Australian dentists record basic personal data of patients in 82% of cases, with only 29% taking additional personal details, which is less often than Croatian dentists [1]. The study by Thampan et al. on a sample of 543 dentists from southern India showed that almost all respondents (97%) record basic patient data; however, the recording of additional data was not investigated [16]. Survey results of American dentists showed similar results to their Croatian counterparts regarding updating information in dental documentation [17]. Most dentists agree on the importance of updating information and cite time constraints as a major obstacle.

Two Indian studies have shown that 89% of dentists in India record dental anomalies and anatomical variations such as the torus mandibularis [16,18], significantly more so than in Croatia. Our results are similar to those in a survey on Australian dentists [1], who keep good records of personal patient data and details of restorative procedures, but somewhat less so when it comes to dental anomalies.

In terms of using abbreviations for recording treatment, our results are comparable to those from Indian dentists, with 67% of dentists using abbreviations or codes [18]. Abbreviations are kept by as much as 64% of Indian dentists as opposed to 30% of Croatian dentists.

In a paper on dental investigation in an air disaster, Ligthelm emphasized the need for international standardization of abbreviations and the maintenance of dental records [10]. This problem is particularly significant in accident investigations involving victims from other countries, as was the case in Thailand after the 2004 tsunami [19]. This is increasingly the case due to the current increasing trend of international migration. In 2019, it was estimated that there are 272 million people living in countries other than their country of birth, which is 3.5% of the world’s population [20]. With approx. 82 million migrants, Europe ranks second place, after Asia which has 84 million migrants. Thousands of migrants die while trying to reach Europe, becoming “missing migrants”. The Mediterranean Sea is where the highest number of known deaths during migration occur, i.e., 17,919 deaths over a span of five years (2014–2018). These trends are increasing the demand for forensic odontology expertise in human identification and age estimation for the purposes of preventing human rights violations [21]. Croatia, an EU member state, is a transit country for migrants that illegally cross EU borders while heading to Western Europe, and at the same time a country of origin for economic migrants who settle in Western European countries. Thus, legible dental documentation of Croatian citizens may be requested by dentists and forensic odontologists in other European countries, while Croatian forensic odontology experts may encounter challenges in obtaining dental records of migrants from Africa and Asia.

Poor record keeping can be expected in less developed countries [16,18,22,23,24], but research shows such insufficiencies even in highly developed countries such as the United Kingdom, where as many as 44% inaccurate dental records were found [25]. Research in Sudan has shown that dental students keep dental records more accurately than dentists in private clinics [23].

The situation is similar with archiving dental records, which is regulated by law in developed countries. It may come as a surprise that the legal obligation to maintain such documentation was introduced in Belgium as late as in 2004, and prior to that, it was only deontological and ethical codes that imposed the obligation on dentists to do so [12]. Dentists in India often do not keep records of treatments performed (22%), and if they keep X-rays, it is only for a few months up to a maximum of three years [18]. In Australia and New Zealand, as many as 85% of dentists keep X-rays taken by other dentists, and 63% retain even faulty X-rays [1]. Both Australian and Indian dentists are exceptionally consistent in recording implant serial numbers (70%) [1,18] and, to a lesser extent, Croatian dentists in our research (61%).

American dentists recognize the importance of adequate record keeping and archiving but, due to the lack of guidelines for updating patient records, they spend more time in other aspects of dental practice [17]. Perceived barriers to making accurate and complete dental records by Australian dentists are increased workloads in practice, time constraints, insufficient space for archives, lack of record quality check personnel, as well as lack of experience and education [1]. In our research, only a third of the dentists were familiar with the legal requirement of retaining documentation, and a fifth of the dentists had no knowledge of forensic dentistry and the possible use of dental documentation in forensic procedures.

In Croatia, forensic odontology was introduced as a mandatory course in undergraduate programs in 1997, with the establishment of the Chair of Forensic Dentistry at the School of Dental Medicine, University of Zagreb. It was later introduced in postgraduate programs and professional continuing education. Throughout the course, participants are introduced to the legal obligations of record keeping (informed consent, diagnosis, treatment plan, recording treatment) and the importance and application of documentation in dental identification and use in forensic expertise and litigation related to negligence, malpractice, and the qualification of orofacial injury. Since 2015, the harmonization of the study curriculum with recommendations of the Association for Dental Education in Europe has led to forensic odontology becoming an elective course in undergraduate programs. This has resulted in only some students enrolling onto the course, which is certainly a step backwards.

The International Organization for Forensic Odonto-Stomatology (IOFOS) investigated undergraduate education in forensic odontology and found that a specific teaching course in forensic odontology is neither mandatory nor elective in most undergraduate programs [26]. At the same time, the profile and competences for the graduating European dentist include the Professionalism domain, composed of ethics, regulation, and professional behavior [27], which are covered in a basic forensic odontology course.

## 5. Conclusions

The results of this research show that Croatian dentists keep and store detailed dental documentation to a great extent; however, there are some insufficiencies in recording dental anomalies and non-carious lesions, as well as omissions in taking and updating dental status, omissions in the use of codes, and inconsistencies in recording fillings, as well as insufficient knowledge of legally required conditions for archiving records. The proper maintenance of dental documentation is become increasingly important due to the development of dental tourism, bringing a large influx of patients from foreign countries. Migration is also causing an increasing demand for forensic odontology expertise, requiring assessments of dental documentation from foreign countries, if available. There is evidently a need for additional education and raising awareness on the importance of properly maintaining dental records. This new direction is necessary not only to facilitate identification of unidentified bodies or bitemark perpetrators, but also to protect dentists from possible lawsuits and litigation. Our research data also indicate the need to adopt a national and international standard for keeping detailed and legible documentation that meets contemporary trends in health care and the requirements of forensic procedures.

Including forensic odontology in compulsory undergraduate courses as well as postgraduate and continuing professional education would equip dentists with knowledge and awareness of the importance and possible application of dental documentation. At the same time, it would allow dentists to achieve and maintain competences in the domain of professionalism. We expect the IOFOS to take further steps in advising authorities to include a basic course of forensic odontology in the undergraduate dental curriculum to meet these goals. Also, dental chambers should be advised to impose a continuing education course on the legal requirements for dental practice as a prerequisite for renewal of the license.

## Figures and Tables

**Table 1:** General information on dentists (number, percentage (%)).

Gender	Female	116 (80.0)
	Male	29 (20.0)
Age	25–45 years	102 (70.3)
	45–65 years	43 (29.7)
Work experience	<5 years	33 (22.8)
	5–20 years	78 (53.8)
	20< years	34 (23.5)
School of basic dental degree	School of Dental Medicine University of Zagreb	118 (81.4)
	Study of Dental Medicine University of Rijeka	16 (11.0)
	Study of Dental Medicine University of Split	7 (4.8)
	Other	4 (2.8)
Practice location, region	Central Croatia	68 (46.9)
	Istria and Croatian Littoral	18 (12.4)
	Slavonia	23 (15.9)
	Dalmatia	36 (24.8)
Type of employment	Public health center	49 (33.8)
	Private practice with CHIF * contract	42 (29.0)
	Private practice	31 (21.4)
	Clinic/polyclinic	23 (15.9)

* Croatian Health Insurance Fund.

**Table 2:** Dental documentation: data items recorded/retained (number, percentage (%)).

Record patients’ basic personal data		145 (100.0)
Record patients’ additional personal details (maiden name, name of general practitioner, name of another dentist)		67 (46.2)
Medical history	Record	135 (93.1)
	Record and update with each visit	93 (64.1)
	Record on first visit	126 (86.9)
Full dental status	Update with each visit	45 (31.0)
	Update twice a year	41 (28.3)
Record additional data on dentition	Changes in dentitions	80 (55.2)
	Trauma data Dental anomalies	119 (82.1)
	Number Position Shape Diastema	87 (60.4)
		48 (33.1)
		16 (11.0)
		19 (13.1)
	Non-carious lesions	51 (35.2)
	Occlusion, Angle’s classification	29 (20.0)
Record details for restorative treatment	Filling surface	104 (71.7)
	Filling material	104 (71.7)
	Other (color, Black’s classification, type of preparation)	87 (60.0)
Use abbreviations/codes for recording treatment		101 (69.7)
Store past list of codes for treatment stipulated by the CHIF after they have been changed		43 (29.7)
Use of tooth coding	FDI system	104 (71.7)
	Palmer–Zsigmondy system	28 (19.3)
	ADA Universal system	20 (13.8)
Routinely take X-rays	Orthopantomogram	137 (94.5)
	Periapical radiograph	101 (69.7)
	Bitewing radiograph	45 (31.0)
	Do not routinely take X-rays	5 (3.4)
Take intraoral or extraoral (facial) photographs		89 (61.4)

**Table 3:** Practice and duration of dental documentation keeping, and barriers to good practice (number, percentage (%)).

Format of dental records	Digital form	135 (93.1)
	Digital form with backup	99 (68.3)
	Paper form	73 (50.3)
X-ray format	Analog	63 (43.4)
	Digital	137 (94.5)
Duration of X-ray keeping	<5 years	18 (12.4)
	5–10 years	54 (37.2)
	11–15 years	34 (23.4)
	16–20 years	9 (6.2)
	>20 years	30 (20.7)
Other documentation keeping up to 10 years	Dental casts	137 (94.5)
	Temporary works	141 (97.2)
	Implant serial number	88 (60.7)
Barriers to good practice of record keeping	Lack of time	110 (75.9)
	Lack of education	43 (29.7)
	Lack of storage space	51 (35.2)
	Do not consider it important	5 (3.4)

**Table 4:** Knowledge of legal aspects of dental practice (number, percentage (%)).

Know the law on record retention for 10 years		47 (32.4)
Obtain written consent before treatment		100 (69.0)
Retain informed consent		102 (70.3)
Consider the patient’s rights in access to information	Right to the original records	33 (22.8)
	Right to a copy of records	102 (70.3)
	No rights	10 (6.9)

**Table 5:** Awareness of forensic odontology (number, percentage (%)).

Recognize the scope of forensic odontology in:	Identification of the deceased in unidentified cases	138 (95.2)
	Identification of the perpetrator by bitemark analysis	109 (75.2)
	Other legal proceedings	97 (66.9)
Familiarity with forensic odontology gained in:	Undergraduate study	104 (72.2)
	Specialist study	5 (3.4)
	Professional continuing education	19 (13.1)
	Doctoral study	12 (8.3)
	No knowledge	32 (22.1)

**Table 6:** Legal requirements for dental documentation maintenance in Croatia.

Requirement	Law/Regulation
Patients’ basic personal data to be registered in e-charts	Regulation on maintenance of electronic personal health record
Dental documentation must be accurate, detailed, and dated, covering patient’s status and treatment	Law on dental medicine
Documentation in electronic form must be protected from changes, unauthorised use, and early destruction	Law on dental medicine
Dental documentation consists of dental record with status chart, medical/dental history, radiographs, and photographs	Law on dental medicine
Obligation to allow patient to access documentation	Law on dental medicine
Obligation of record retention for 10 years	Law on dental medicine
Patient’s right to informed consent Content of informed consent form/refusal form	Law on patients’ rights Law on informed consent/refusal form
